# ROS Metabolism Perturbation as an Element of Mode of Action of Allelochemicals

**DOI:** 10.3390/antiox10111648

**Published:** 2021-10-20

**Authors:** Pawel Staszek, Urszula Krasuska, Katarzyna Ciacka, Agnieszka Gniazdowska

**Affiliations:** Department of Plant Physiology, Institute of Biology, Warsaw University of Life Sciences, Nowoursynowska 159, 02-776 Warsaw, Poland; urszula_krasuska@sggw.edu.pl (U.K.); katarzyna_ciacka@sggw.edu.pl (K.C.)

**Keywords:** phytotoxicity, allelopathy, specific reactive oxygen species (ROS), antioxidants, *meta*-tyrosine, allelochemicals mode of action, protein nitration

## Abstract

The allelopathic interaction between plants is one of the elements that influences plant communities. It has been commonly studied by applying tissue extracts onto the acceptors or by treating them with isolated allelotoxins. Despite descriptive observations useful for agricultural practice, data describing the molecular mode of action of allelotoxins cannot be found. Due to the development of -omic techniques, we have an opportunity to investigate specific reactive oxygen species (ROS)-dependent changes in proteome or transcriptome that are induced by allelochemicals. The aim of our review is to summarize data on the ROS-induced modification in acceptor plants in response to allelopathic plants or isolated allelochemicals. We present the idea of how ROS are involved in the hormesis and plant autotoxicity phenomena. As an example of an -omic approach in studies of the mode of action of allelopatic compounds, we describe the influence of *meta*-tyrosine, an allelochemical exudated from roots of fescues, on nitration—one of nitro-oxidative posttranslational protein modification in the roots of tomato plants. We conclude that ROS overproduction and an induction of oxidative stress are general plants’ responses to various allelochemicals, thus modification in ROS metabolisms is regarded as an indirect mode of action of allelochemicals.

## 1. Introduction

### Allelopathy—A Brief History of Publication concerning the Phenomenon

According to Charles Darwin’s theory of the struggle for existence, higher plants compete with each other in the ecosystem for light, water, and nutrients. As a result of this competition, plants have developed various strategies to conquer their neighbors. In all cases in which this protection or weapon is of chemical nature, it is called allelopathy. Although this phenomenon was described in the past, the term allelopathy was only proposed by Hans Molisch in 1937 in a book entitled *Der Einfluss einer Pflanze auf die andere: Allelopathie*. The word “allelopathy” derives from Greek: “allelon” means “mutual” or “among each other”, and “pathos” means “suffering” or “feeling”. Despite the key importance of Molisch’s work for the study of interactions between plants, his research is distant from the modern-day understanding of the topic of allelopathy. For instance, Molisch studied the effect of ethylene, which is currently considered as a phytohormone, not an allelochemical [1]. A century before the publication of Molish’s book, in 1832, Augustin Pyrame de Candolle laid the foundations for a scientific view on plant–plant and plant–soil interactions. The Swiss botanist stated that the substances secreted by plants are the main factor for yield decline in crops, and named the phenomenon soil sickness [2].

**Figure 1:**
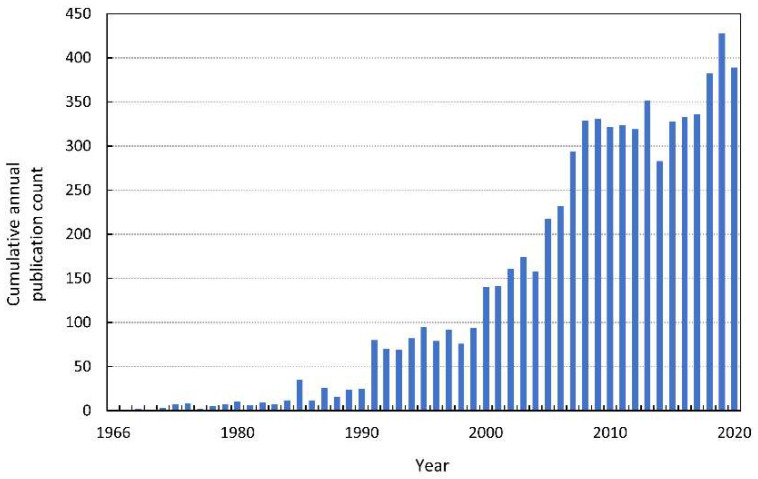
The cumulative annual publication count of papers on allelopathy. Details of the search are described in the Supplementary Materials.

In 1966, the first modern publication on allelopathic interactions was published. The paper—“The role of chemical inhibition (allelopathy) in vegetational composition”—published by Cornelius Muller is also the first article with the term “allelopathy” noted in the Web of Knowledge (Figure 1). This work is an example of a methodological approach to allelopathy that dominated in the second half of the XX century. The author performed bioassays for volatile compounds, extracted compounds responsible for allelopathic effects from donor plants, and finally identified them. Another milestone in allelopathy research was undoubtedly the book by Elroy L. Rice, *Allelopathy*, published in 1974. A decade after this work, the number of articles listed in the Web of Knowledge increased from a few per year to a dozen, and quickly to several dozen per year (see Figure 1 for search details in the Supplementary Materials). In 1996, the International Allelopathy Society (IAS) defined allelopathy as “any process involving secondary metabolites produced by plants, algae, bacteria and fungi that influences the growth and development of agriculture and biological systems, excluding animals”, which coincides with the year in which the number of published papers on allelopathy reached almost 100 [1].

Reactive oxygen species (ROS) are by-products of an aerobic metabolism and are formed as a result of an incomplete reduction of molecular oxygen. ROS includes, among others, superoxide anion radical (O_2_^•−^), hydroxyl radical (^•^OH), and hydrogen peroxide (H_2_O_2_) [3]. ROS are produced in various cell compartments: plastids, mitochondria, peroxisomes, and in biological membranes. The effect of ROS (depending on their concentration) is usually associated with disturbances in redox homeostasis and the induction of oxidative stress, which is nowadays defined as “an imbalance between oxidants and antioxidants in favour of the oxidants, leading to a disruption of redox signalling and control and/or molecular damage” [4]. Oxidative modifications of lipids, proteins, and nucleic acids may lead to cell death [5]. Under physiological conditions, the formation and scavenging of ROS are precisely controlled, but this balance can be disturbed by stresses. Aerobic organisms evolved an effective system of ROS neutralization. ROS could be scavenged due to the activity of enzymes, such as catalase (CAT), superoxide dismutases (SOD), glutathione peroxidase (GPx), glutathione reductase (GR), ascorbate peroxidase (APX), and class III peroxidases (POX). The enzymatic antioxidant system is supported by low-molecular weight compounds, e.g., glutathione (GSH), ascorbate, α-tocopherol, carotenoids, and phenolic compounds [6].

**Figure 2:**
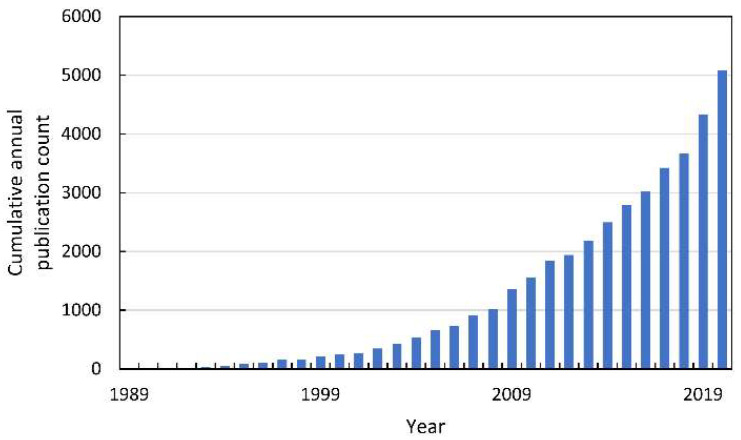
The cumulative annual publication count of papers on oxidative stress in plants. Details of the search are described in Supplementary Materials.

In the last decade of the XX century, oxidative stress in plants became the subject of intense research, which resulted in the publication of thousands of reports (Figure 2).

**Figure 3:**
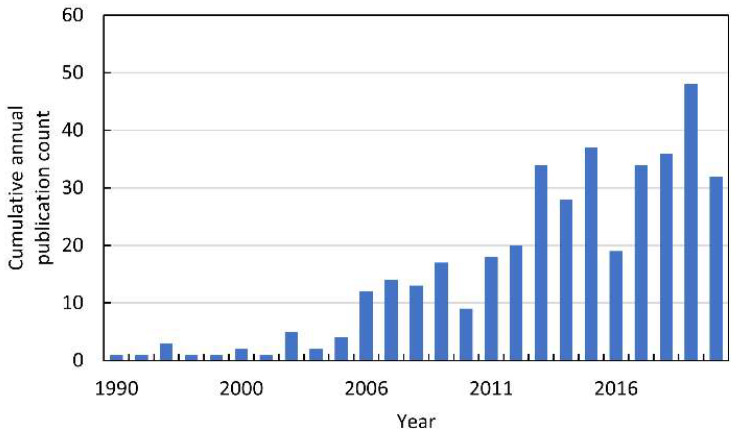
The cumulative annual publication count of papers on ROS in allelopathy. Details of the search are described in Supplementary Materials.

In the 1990s, the first papers linking allelopathy with ROS were published (Figure 3). However, these were just single publications that referred to the phenomenon of plant–insect interactions [7,8,9,10,11]. It is worth adding that these works were published before the exclusion of animals from the definition of allelopathic interactions. Until 2015, 132 papers indexed in Web of Knowledge were published on ROS in allelopathy. Most of them were summarized in our previous extensive review [12]. Since the publication of this chapter, the number of papers linking ROS to allelopathy has almost doubled (Figure 3).

Allelochemicals are products of a secondary metabolism, thus they are considered as unnecessary for cell development. They are released by a donor plant into the environment *via* volatilization, leaching, root exudation, or decomposition of plant residues, which is the greatest source of allelotoxins. A release of allelochemicals by root exudation was considered marginal in previous research; however, nowadays, it is believed to be more important because 5 to 21% of fixed carbon could be excreted by roots [13]. Allelochemicals may be localized widely among organs, such as roots, stems, leaves, flowers, pollen, or seeds; sometimes toxic compounds are found in only one or two organs. Production of allelotoxins is not widespread in the plant kingdom; a particular group of allelochemicals is synthesized by a restricted range of plant families; for instance, hydroxamic acids are characteristic for Poaceae [14].

Since the beginning of the study of allelopathic interactions, numerous compounds of strong allelopathic potential were identified and characterized. Allelochemicals are chemically diverse and represented by terpenoids (monoterpens, diterpens, triterpens, sesquiterpens, and steroids), phenolic compounds (simple phenolics, coumarins, quinones, and flavonoids), nitrogen-containing chemicals (alkaloids, non-proteinogenic amino acids, cyanogenic glycosides, and benzoxazinoides) and others. Due to the great chemical diversity of allelotoxins, it is impossible to point one or even several pathways of their biosynthesis. However, most of the well-characterized allelochemicals are formed through the acetate and shikimate pathways [15].

Allelopathy is a complex phenomenon with a web of connections with many ecological and physiological processes. Research conducted in laboratory conditions on whole plants, their fragments, tissue cultures, and with the use of plant extracts, single compounds or synthetic equivalents isolated from them is often referred to as the “phytotoxic effect” or “phytotoxicity”. Determining phytotoxicity should be the first step in the investigation of allelopathy interactions, but it is not enough to conclude that the allelopathic interaction occurs in the ecosystem [16].

## 2. The Allelopathic Potential of a Plant Reflects Disturbances in the Redox State of the Acceptor Plant—Experiments with Application of Plant or Tissue Extracts Serve as an Introduction to Studies on the Mode of Action of Isolated Allelochemicals

**Figure 4:**
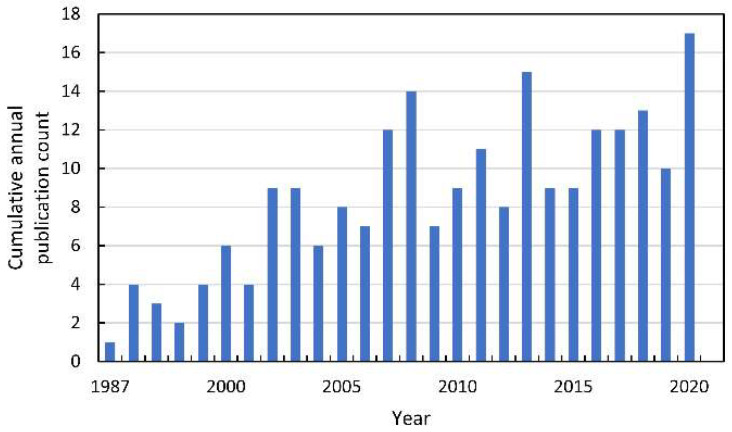
The cumulative annual publication count of papers on the mode of action of allelochemicals. Details of the search are described in Supplementary Materials.

Since the beginning of the XXI century, an increase in the number of publications referring to the mode of action (MOA) of allelochemicals in acceptor plants has been observed (Figure 4). Some of the most preliminary data are collected after studying the impact of extracts from the organs of allelopathic plants, exudates, or debris tissues used as a mulch. Therefore, there is a considerable number of studies describing the induction of oxidative stress after the application of the mixture of the chemicals, e.g., in water extracts prepared from the tissue of allelopathic plants. This methodological approach, to some extent, corresponds to the allelopathy phenomenon that can be seen in the natural environment or agroecosystems, in which allelopathic plants influence neighboring organisms by the combination of chemicals, not only by a single phytotoxin. One of the first reports pointing to the link between allelopathy stress and secondary oxidative stress described observations made on mustard (*Sinapis alba* L.) seeds, whose germination was inhibited by the water extracts of sunflower (*Helianthus annuus* L.) leaves [17]. Authors suggested that the restriction in germination of mustard seeds was linked to the increased level of ROS, which were not sufficiently scavenged by an antioxidant enzymatic system (CAT, SOD, and GR). Similar data were shown in tomato (*Solanum lycopersicum* L.) roots after a short term treatment with the extract of *Sicyos deppei* G. Don, an invasive annual tendril-bearing vine endemic to Mexico [18,19]. In this case, ROS were indicated as signals that activated a cascade of other events leading to cell malformations. Such descriptive data were also reported after the determination of the allelopathic potential of aqueous extracts (1.0–8.0%) of shoots of the knight’s milfoil (*Achillea santolina* L.), a common weed in wheat plants (*Triticum aestivum* L.) crops in the Middle East region of Asia and in Europe [20]. Extracts of *A. santolina*, in a concentration-dependent manner, inhibited the germination of wheat kernels, restricted seedling growth, and led to chlorophyll degradation. It was accompanied by disruption in membrane integrity, demonstrated as increased electrolyte leakage and high malondialdehyde (MDA) levels. An increase in lipid peroxidation was correlated with elevated levels of H_2_O_2_, suggesting disturbances in the antioxidant properties of wheat tissues under allelopathic stress originated from chemicals of *A. santolina* shoots. Although in wheat leaves exposed to extracts of *A. santolina* SOD, APX, GPX, and GR activities were generally stimulated, CAT activity was restricted. APX and GR activity was correlated to a high GSH/GSSG ratio, suggesting a significant role of the oxidative stress in the MOA of the mixture of allelochemicals in water extracts from shoots of *A. santolina* [20].

In the last 10 years, many more studies on oxidative stress as a MOA of allelochemicals were conducted in the context of the importance of allelopathy for the regulation of crop productivity and alternative weed management methods. Several attempts have been made to compare the effect of an application of the extracts obtained from tissues of an allelopathic plant to the effect of a group of phytotoxins or single phytotoxin, identified in the extracts or root exudates. Sorghum (*Sorghum bicolor* L. Moench) plants in the form of sorgaab as a source of strong allelochemicals (sorgoleone and some phenolics) are commonly used in many countries in Asia for weed control ([21] and references in [21]). The MOA of one of the well-recognized allelochemicals—sorgoleone, which is present in root exudates of sorghum plants—is linked to the inhibition of the electron transport chain in mitochondria and chloroplasts, resulting in a decrease in the mitochondrial respiration rate and PSII inhibition ([22], references in [22,23]). Novakoski et al. [24] demonstrated that an inhibition of oxygen uptake by water extracts of sorghum roots or stems in milkweed (*Euphorbia heterophylla* L.) is accompanied by an increased activity of CAT and POX, suggesting the induction of oxidative stress. Rattle weed (*Crotalaria retusa* Linn) is an annual plant that grows very rapidly and suppresses nearby plants. Ogunsusi et al. [25] investigated the inhibiting potential of an alkaloid fraction of *C. retusa* on common bean (*Phaseolus vulgaris* L.) seedlings. They observed increased CAT and SOD activity in stems and leaves of bean plants, depending on the concentration of the *C. retusa* alkaloid fraction (50–100 µg mL^−1^). It was accompanied by an increase in the content of GSH and ascorbic acid, suggesting that oxidative stress resulting from an overproduction of ROS could be the basis of metabolic reprogramming related to the toxicity of alkaloids produced by *C. retusa*.

Some authors examined the possibility of using aromatic plants, which produce essential oils (EOs), or EOs as a source of bioherbicides against frequently occurring weed species. Dmitrović et al. [26] investigated the phytotoxic potential of EOs (containing mostly nepetalactones, accompanied by ß- or α-pinene and other monoterpenes) of catmint (*Nepeta rtanjensis* Diklić and Nikojević, and *Nepeta cataria* L.) on common ragweed (*Ambrosia artemisifolia* L.). EOs from both catmint species inhibited the growth of ragweed roots and shoots, and led to a discoloration of the shoots. Monoterpenoids are known to induce the overproduction of ROS, particularly H_2_O_2_ [27]. Thus, alterations in the activity of enzymatic antioxidants in in vitro cultured ragweed shoots were not surprised. CAT and SOD activities were decreased after 2 weeks of being exposed to EOs, while POX activity was slightly higher [26]. Redstem wormwood (*Artemisia scoparia* Waldst. & Kit.) is an annual aromatic herb that inhabits cultivated and uncultivated lands, including wastelands. The plant is used in traditional medicine due to the wide spectrum of EOs produced by the leaves. Artemisia oil (0.14–0.70 mg mL^−1^) induced all the classical symptoms of oxidative stress in the roots of the wheat plants: the increased levels of ROS (H_2_O_2_, O_2_^•−^), loss of membrane permeability, and stimulated lipid peroxidation [28]. The activities of the scavenging enzymes (SOD, CAT, GR, POX, and APX) were significantly elevated, suggesting their action as a secondary defense mechanism in ROS scavenging [28]. The EOs from romerillo (*Hetherothalamus psiadioides* Less.) (1–5 µL) negatively affected the rooting of Arabidopsis (*Arabidopsis thaliana* (L.) Heynh.) microcuttings and induced the whitening of the leaves of the explants [29]. A defective formation of adventitious roots was explained by the induction of oxidative stress, mainly the increased level of H_2_O_2_, which could interrupt the auxin transduction pathway [29]. Another aromatic plant—peppermint (*Mentha x pipera* L.)—has a worldwide distribution and is known due to its allelopathic potential, linked particularly to its high synthesis of OEs (menthol (35%), mentone (17.5%), menthofuran (11.7%), and 1,8-cineole (5.9%))and of phenolic compounds [30]. Among the phenolics extracted from the aerial part of the peppermint plant, the most abundant are trans-ferulic acid, ellagic acid, sinapic acid, and chlorogenic acid [31]. Extracts (2–10% *v*/*v*) rich in phenolics inhibited the seed germination and growth of radish (*Raphanus sativus* L.) seedlings [31]. Detailed analysis of the antioxidant cellular system activity of radish seedlings in response to peppermint water extracts indicated a decreased activity of SOD, and no impact on activities of CAT, POX, or APX; only the total accumulation of phenolics was detected. Such accumulation of phenolics as antioxidants in response to allelopathic stress is often reported. The aqueous extracts of the leaves of some Cupressacea species (*Tetraclinic articulata* (Vahl) Mast., *Juniperus phoenicea* L., and *Cupressus sempervirens* L.), rich in mono- and sesquiterpenes, increased the concentration of flavonoids and tannins, and stimulated the activity of phenylalanine ammonia lyase (PAL) and tyrosine ammonia lyase (TAL) in lettuce (*Lactuca sativa* L.) plants [32]. Although the growth of plants cultured in allelopathic extracts was inhibited, the chlorophyll content was decreased, electrolyte leakage was elevated, and the MDA level was not increased [32], suggesting that accumulated phenolics act as efficient scavengers of ROS. This effect contrasts with the observation made by García-Sánchez et al. [33], who used the aqueous extract of dry olive mill residue (ADOR)— which is of potential interest for use as a fertilizer, but contains a significant amount of phenolics—to investigate its effect on tomato roots. The analyses of the ADOR phenolics composition showed *p*-tyrosol, protocatechuic, and vanillic acids as main components, with *p*-coumaric, gallic, syringic and caffeic, and ferulic and hydroxybenzoic acids occurring in lower concentrations. *In vivo* visualization of O_2_^•−^ and H_2_O_2_ in tomato roots treated with ADOR indicated an accumulation of ROS. An enhanced generation of ROS in the roots of acceptor plants in response to ADOR was associated with an increased activity of SOD, GR, or glutathione S-transferase (GST), and was accompanied by a high content of phenolic compounds. It could be possible that, in this case, phenolics act as main antioxidants, because they are commonly accepted to contribute to antioxidant capacity of the tissue, which was highly elevated in tomato roots after the ADOR treatment [33]. In a similar manner to its effects on tomato plants, ADOR induced an oxidative burst in sunflower roots, with a marked stimulation of O_2_^•−^ production and increased SOD activity [34]. The high level of MDA in sunflower roots treated with ADOR suggests that the antioxidant enzymatic system (mainly SOD), although induced, did not completely eliminate the generated ROS. In addition, POX activities were diminished, which also explains the accumulation of H_2_O_2_.

An elegant work was performed by Szwed et al. [35] on the influence of common buckwheat (*Fagopyrum esculentum* Moench) root residues (BRR) on selected weed species (barnyard grass (*Echinochloa crus-galli* L.), wind grass (*Apera spica-venti* L.), cleavers (*Galium aparine* L.), and tiny vetch (*Vicia hirsuta* L.). The allelopathic potential of buckwheat on other plants has been known for many years, due to various phenolic acids, flavonoids, alkaloids, and fatty acids produced in its tissue and found in the exudates [36,37]. BRR inhibited the growth of barnyard grass and cleavers, while no harmful effect was observed for wind grass and tiny vetch. In all acceptor plants, BRR increased the total content of phenolics and flavonoids. The authors suggested that the increase in the concentration of phenolic acids and flavonoids in the leaves of weeds that are not sensitive to BRR may reflect the induction of oxidative stress. However, the fact that no inhibition of growth was found in wind grass and tiny vetch could be explained by the defense mechanisms of these species, which appears to be more effective than those of barnyard grass and cleavers. Total antioxidant capacity in leaves of barnyard grass and cleavers increased as plants were treated with BRR, which corresponded to the level of phenolic compounds in their tissues. What is more, stimulation of POX activity was also detected in barnyard grass grown in the soil containing BRR, suggesting the overproduction of ROS [35].

Although studies of the allelopathic potential of various plants have been very popular in recent years, many of them are only descriptive. Many authors still only use plant extracts or extracts from plant organs without any trials to isolate individual chemicals. This model partly reflects the natural conditions of the environment, in which plant–plant interactions depend on a mixture of various compounds, but it does not facilitate the understanding of the biochemical and molecular basis of allelopathy.

## 3. Phytoselective Activity of Some Allelochemicals Is Linked to a Species Dependent Modification of ROS Metabolism

### 3.1. Autotoxic Action of Chemicals Depends on the Regulation of ROS Production and Scavenging in Responsive Plants

Allelochemicals are known to be involved in autotoxicity and soil sickness. This phenomenon is a special kind of allelopathy in which allelochemicals produced by the donor plant are toxic for the plant of the same species. Cucurbitaceous plants are known for their high autotoxic potential, although differences in the autotoxicity between genotypes can be observed. The inhibition of growth of cucumber (*Cucumis sativus* L.) or watermelon (*Citrullus lanatus* (Thunb.) Matsum. & Nakai) in monocropping is mostly related to the benzoic and cinnamic acids that can be identified in exudates of their roots [38]. But a figleaf gourd (*Cucurbita ficifolia* Bouché), belonging also to cucurbits, is not sensitive to the exudates of cucumber or watermelon [39]. Cinnamic acid (0.05–0.25 mM, corresponding to about 0.1 mM of the chemical in soils rich in cucumber plant residues) inhibited the growth of roots and shoots of cucumber seedlings, but had no effect on the growth of figleaf guard plants [40]. Cinnamic acid induced the accumulation of ROS (H_2_O_2_ and O_2_^•−^) in cucumber, but not in figleaf gourd roots. It was correlated to an increased activity of NADPH oxidase that was noticed only in cucumber roots, which may be responsible for the over-production of ROS. The activities of SOD, GPX, and APX in the roots of the figleaf gourd were higher than in those of the cucumber in the absence of cinnamic acid and remained at the same level during the treatment, in contrast to the enzymatic (particularly SOD and GPX) activity in the roots of the cucumber, which increased proportionally to cinnamic acid concentration [40]. Thus, in figleaf gourd roots, cinnamic acid induced little changes in ROS generation and ROS-scavenging activity, resulting in the maintenance of membrane integrity and high cell viability, which could be the main explanation for the low sensitivity of the plant to autotoxic allelochemicals. *p*-Hydroxybenzoic acid, a derivative of benzoic acid, was also investigated as an autotoxic compound of cucumber seedlings in the context of oxidative stress [41]. *p*-Hydroxybenzoic acid at a 0.1–1.25 mM concentration inhibited the root growth of cucumber seedlings, although the effect was dependent on cucumber accessions, e.g., Zhongnong 16 (ZN16) and Hexin 25 (HX25). The growth of the roots of ZN16 was much more restricted by *p*-hydroxybenzoic acid than the growth of the HX25 roots. Although *p*-hydroxybenzoic acid increased the expression of the POX, CAT and metallothionein (MT) genes in both the ZN16 and HX25 roots, the transcript levels in the HX25 plants were significantly lower than those in the more sensitive ZN16. Consistently with the lower expression levels of POX, CAT and MT genes in the resistant HX25 plant, it accumulated higher levels of H_2_O_2_ in the root tip than in the ZN16 plant after the application of the autotoxin, which is necessary for the proper growth of root tips. This suggests that the ROS-scavenging capacity could play a key role in the modulation of root growth in the cucumber autotoxic response [41]. Some other examples of chemicals crucial for autoallelopathy, with MOA linked to the modification in ROS metabolism, are included in Table 1. Autotoxicity is a significant problem in closed hydroponic systems, in which the accumulation of allelochemicals in culture solutions results in severe morphological malformations. This phenomenon is commonly observed in the hydroponic culture of cucumber, tomato, or strawberry (*Fragaria* × *ananassa* Duch.), and documented for leafy vegetables, e.g., lettuce ([42] and references in [42]). In a non-renewed nutrient solution used in hydroponics, various phenolic acids and their derivatives were detected [42]. Lettuce plants growing in a non-renewed medium exhibited the typical symptoms of oxidative stress: high levels of ROS (H_2_O_2_ and O_2_^•−^) and high MDA content pointing to membrane lipid peroxidation. Accumulated ROS were not sufficiently scavenged by the cellular antioxidant system, because the activities of SOD, CAT, APX, and POX were inhibited [42]. It was suggested that the activation of enzymatic antioxidants is strongly dependent on the concentration of allelochemicals or phytotoxins. Lower doses of toxins increase the activity of SOD, POX, or APX, and sometimes of CAT, while allelochemicals at a high, harmful concentration decrease the activity of enzymatic antioxidants. This results in the activation of defense mechanisms against cell death after the treatment with allelochemicals at a low dose (hormesis) or in inhibitory and irreversible modifications in the growth and metabolism of the acceptor plant, after its exposition to the compound at a higher concentration.

### 3.2. Hormesis—ROS Act as Signals in the Positive Response of the Acceptor Plant to Allelochemicals or Neighboring Allelopathic Plants

Some allelochemicals, particularly at a low concentration, can have a beneficial effect on the growth of plants that are located in the nearest neighborhood. This phenomenon is known as hormesis. The term refers to a biphasic dose–response to an environmental agent characterized by a low dose stimulation or a beneficial effect and a high dose inhibitory or toxic effect [79]. Aqueous extracts of garlic (*Allium sativum* L.) bulbs (AGE, 50–200 µg mL^−1^), as a foliar application or in the form of fertigation, stimulated the growth of tomato seedlings [80]. The AGE also had effects on the activity of POX and SOD, which were stimulated. This reflects the induction of defense mechanisms against oxidative stress, and AGE may be used as priming agent in this situation.

Very interesting experiments were performed on a co-culture of garlic and pepper (*Capsicum annum* L.) plants in a hydroponic system [81]. The ratio of the pepper to garlic of 1:1–1:2 had a stimulating effect on the growth of the pepper plants, whereas at the ratio of pepper to garlic of 1:4 or 1:6 a decline in chlorophyll content and an inhibition of shoot and root growth were observed. Morphological changes were associated with the twofold increase in the MDA level in the pepper in the co-culture at the ratio of pepper to garlic of 1:6. In such conditions, CAT and SOD activities were inhibited, whereas lower garlic amounts in the co-culture resulted in the stimulation of CAT and POX activities in the pepper. The beneficial effects of garlic root exudates (ratio of pepper to garlic at the range of 1:1–1:2) was suggested to be linked to the presence of phenolic compounds, which may directly scavenge ROS, minimize membrane deterioration [81], and act as protective agents.

Allicin produced by green garlic is widely known as fungicide used in modern organic agriculture. There are also some reports of the allelopathic action of volatile organic compounds (VOCs) of green garlic, particularly of diallyl disulfide (DADS). Yang et al. [82] investigated the action of VOCs derived from green garlic and DADS applied alone in the regulation of ROS metabolism in the leaves of cucumber plants. DADS, at a concentration of below 1 mM and fumigation with VOCs volatilized from 2 g of green garlic (corresponding to around 1 mM of DADS), had no harmful effect on the morphology of the cucumber leaves, whereas garlic volatiles and DADS at a higher concentration caused the softening and rotting of the leaves, preceded by the yellowing of the tissues due to chlorophyll degradation. To investigate the impact of garlic volatiles and DADS on ROS formation and the activity of the enzymatic antioxidant cellular system in the acceptor plant, the authors co-cultured 12 cucumber seedlings with 0, 6, 12, or 18 garlic seedlings or sprayed cucumber plants with 5 mL of 1 mM DADS [82,83]. Both VOCs and DADS treatments decreased the O_2_^•−^ content in the cucumber leaves and increased the H_2_O_2_ levels. The SOD activity was higher only in co-culture experiments with 18 seedlings of green garlic, whereas CAT activity was slightly lowered. In contrast, POX activity increased when cucumber seedlings were co-cultured with green garlic, and this effect was more pronounced than after the DADS foliar spray. The alterations in activity of antioxidant enzymes did not correspond to their gene expression level. The authors concluded that elevated levels of H_2_O_2_ could act as a signal that activates putative disease resistance in cucumber seedlings. A hormetic effect was observed also for juglone at a lower (± 30 µM) concentration in mustard (*Sinapis alba* L.) seedlings, but only in plants stressed with 10% methanol [84]. Thus, it was suggested that juglone may act as a scavenger of ^•^OH in acceptors stressed by highly oxidative agents, while in less anxious conditions, rather, the pro-oxidative activities of juglone are exhibited [84].

### 3.3. The Invasiveness of Some Plants May Be Linked to the Production of Phytotoxins That Disturb the ROS Metabolism in Native Species

At the beginning of XXI century, several controversial studies were conducted on the invasiveness of spotted knapweed (*Centaurea stoebe* Lim.) and (±)-catechin action as an allelochemical responsible for the expansion of the plant in North America [85,86,87,88]. Since then, although the question on the (±)-catechin role in accessions was not definitely solved, there has been some evidence that many exotic invasive species benefit from allelopathy by their phytotoxins. The novel weapon hypothesis, pointing at allelochemicals produced by invader plants as a key element of their success against native species, was proposed by Callaway and Ridenour [89], and later developed by other researchers [90,91,92,93,94]. Although the history of the study of allelopathy as a beneficial tool used by invasive plants on native plants [95,96] is long, the understanding of the MOA of phytotoxins responsible for the phenomenon still needs exploration. Kalisz et al. [97], in their bioinformatics work, identified 524 invasive plant species across 3 datasets: 81 impactful invasive plants identified in Pysek et al. [98], 19 species from Zhang et al. [99], and 330 from the International Union for Conservation of Nature (https://www.iucn.org/) list of global plant invaders. Species were categorized as allelopathic if a direct or indirect effect of the allelochemical was reported in at least one site where the invader was studied. Allelopathy occurred in more than 50% of the invasive species in their database, pointing to the fact that allelopathy is a widespread trait of successful invaders and explains an important component of the biotic impacts on native plants and their associated beneficial microbes in invaded communities [97].

Regardless of the discussion on the MOA of catechin, which was suggested to induce oxidative stress in acceptor plants due to the elevation of ROS production, e.g., in Arabidopsis roots [88,100], there are some other reports linking invasiveness to allelochemicals and secondary oxidative stress induced by allelochemicals.

The common reed (*Phragmites australis* (Cav.) Trin. ex Steud.) is considered as the most invasive plant in marsh and wetland communities in the eastern part of the United States. Root exudates of two *P. australis* genotypes, BB (native) and P38 (an exotic), were tested for phytotoxicity on different plant species: Arabidopsis, tobacco (*Nicotiana tabacum* L.), turnip (*Brassica rapa* var. *rapa*), and lettuce. Exudates of an exotic *P. australis* line P38 were much more toxic than exudates of the native BB line [58]. In the exudates of the P38 line, a high level of gallic acid (30–80 µmol g^−1^ FW soil) was found, which was correlated to the invasiveness of the plant. The authors demonstrated that the toxicity of gallic acids is linked to the elevated level of ROS. Gallic acid (50 µM) inhibited growth of Arabidopsis roots and altered root morphology (Table 1). The tested allelochemical induced a ROS wave on the root surface, which was associated with the reorganization of cortical microtubules in the cells. Microtubules were diffused and single fibers were gathered into local aggregates [58]. Such undirected reorientation of microtubules as was observed in the case of gallic acid, leading to disorder in cellulose deposition, disturbed elongation growth and resulted in the collapse of roots [58].

The alligator weed (*Alternanthera philoxeroides* (Mart.) Griseb.) is a perennial invasive species in China. It originated in Brazil, but was introduced to Shanghai in the early 1930s, and, nowadays, it is considered as one of the most dangerous invasive alien species in China. The invasiveness of the alligator weed was linked to the allelochemicals present in water extracts of the leaves, roots, or stems. Huang et al. [101] investigated the impact of the alligator weed on the growth of *Zoysia matrela* (L. Merr., commonly known as Manila grass) and identified ethyl propionate as the most abundant component in root extracts of this invasive plant. The authors demonstrated that the effect of ethyl propionate on the SOD and CAT activities of the Manila grass ranged from slightly stimulatory (at a concentration lower than 0.5 mM) to highly inhibitory (at a concentration higher than 1 mM), displaying a similar pattern to the effects of *A. philoxeroides* extracts [101]. Moreover, an increased content of MDA was noticed in Manila grass plants both after treatment with the alligator weed extracts and after exposition to ethyl propionate. This data suggest that modification of ROS metabolism, lipid peroxidation, and membrane deterioration may explain the toxicity of the tested invasive plant. Although, as was suggested by the authors, more detailed studies are necessary to prove the hypothesis, a similar situation occurs reagrding mile-a-minute weeds (*Mikania micrantha* Kunth.) and *Ipomoea cairica* (L.) Sweet., two of the most invasive plants in Hainan island, a tropical province of China. Both plants present perennial twining lianas with similar ecological niches. *M. micrantha* is native to South and Central America, wheres the native range of *I. cairica* is uncertain. Beside their physiological features (big leaves of high photosynthetic rate) that may be crucial for the invasiveness of the species, both plants produce strong allelochemicals [102]. The most abundant allelochemical found in the leaf extracts of the *M. micrantha* is benzoic acid, while cinnamic acid was identified in the leaf extract of the *I. cairica* plant [102]. Both allelochemicals (at a concentration of 10–200 mg mL^−1^), similarly to water extracts of leaf of *M. micratha* or *I. cairica* (50–400 mg mL^−1^) inhibited the growth of the seedling of garland chrysanthemum (*Glebionis coronaria* (L.) Cass. ex Spach., formerly called *Chrysanthemum coronarium*), which is used as a popular leaf vegetable in China. Identified phenolic acids increased the MDA content in garland chrysanthemum, suggesting increased lipid peroxidation, which was associated with the stimulation of CAT and SOD activity particularly at a concentration of cinnamic and benzoic acids at the range of 10–100 mg mL^−1^ [102]. It should be noticed that the negative effect of cinnamic acid on the membrane permeability of garland chrysanthemum was stronger than that of benzoic acid during the first three days of the experiment, which could, in part, explain the higher toxicity of *I. cairica* leaf extracts toward the tested acceptor plant.

Some examples of plants characterized by their high allelopathic potential and classified as invasive species were described by Wang et al. [103], although there is still a lack in comprehensive studies on the link between the invasiveness of the plant, the produced allelochemicals, and their MOA related to the ROS generation or scavenging.

## 4. Induction of Oxidative Stress as a Secondary Mode of Action of Phytotoxins

**Figure 5:**
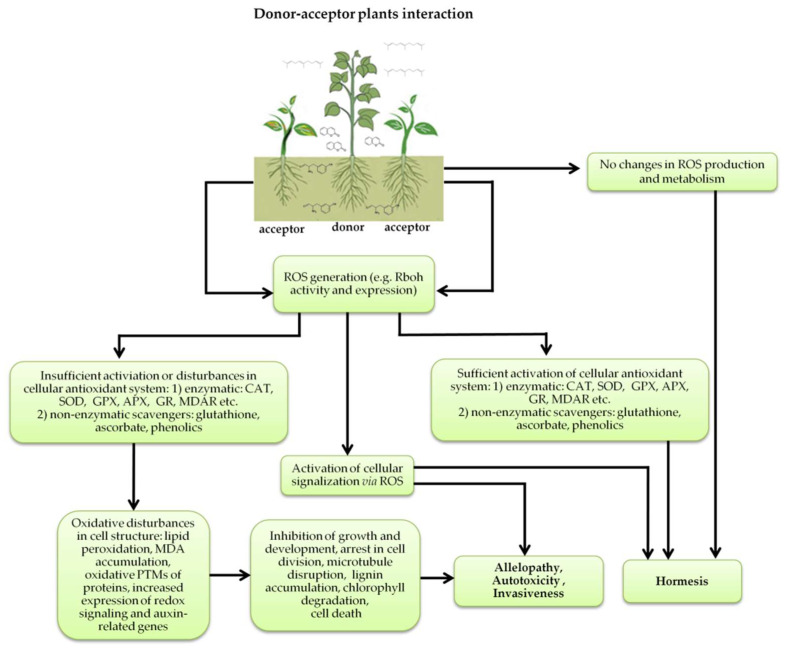
Donor—acceptor plant interaction via allelochemicals results in allelopathy, authotoxicity, invasiveness, or hormetic effect. Phytotoxins may induce ROS generation and activate the antioxidant system leading to ROS scavenging and ROS signalization. Depending on the ROS concentration, the hormetic effect or destructive effects could be noticed. At a high concentration of ROS due to ROS overproduction and disruption in the antioxidant system in susceptible acceptor plants, allelochemicals induced oxidative stress which was manifested as typical oxidative damages of cell structures, resulting, finally, in cell disintegration and death. *meta*-Tyrosine represents allelochemicals present in root exudates: coumarins as leaching allelochemicals and farnesene as volatiles.

The increasing amount of publications on the MOA of various allelochemicals indicates a clear relationship between the toxicity of the chemical and disturbances in the redox metabolism of the acceptor plant (Table 1). Beside a very simple observation of the negative effects of allelochemicals on the growth, development, and morphology of the target organisms, many authors try to correlate the toxicity of the chemicals with their impact on ROS metabolism. Thus, the level of ROS and the modification of the activity of the cellular antioxidant system seem to be useful markers of plant response to allelochemicals. As ROS are considered to be signaling molecules acting as key regulators of basic metabolic pathways in plants, disturbances in redox homeostasis leading to increased ROS levels result in damages of cellular structures and alterations in fundamental physiological processes (Figure 5). A higher sensitivity of the root to phytotoxins (in comparison to shoot) seems to be a typical reaction of the model plants, independently of the structure of the allelochemical (Table 1). Most of the allelochemicals whose MOA was studied to date act as inhibitors of root growth and stimulators of cell death, e.g., coumarin, farnesene, chalcone, isoliquiritigenin, and ß-cembrenediol. The imbalance of ROS production and scavenging in plants exposed to allelochemicals (e.g., BOA, juglone, and monoterpenes, such as ß-pinene ß-myrcene, and thymol) usually corresponds to increased membrane permeability, which is shown as the accumulation of MDA or increased lipid peroxidation. The destructive action of many phytotoxins belonging to different groups of chemical compounds (citral, cembrenediol, umbelliferone, cyanamide, gallic acid, and farnesene) is manifested by an arrest in cell division and microtubule disruption, resulting in the malformations of organ growth. A thickening of the cell wall, particularly in root tips, correlated to lignin accumulation, is often observed as a plant response to various allelochemicals (e.g., ferulic acid) leading to severe abnormalities of morphology. Some allelochemicals are well known as inhibitors of photosynthesis, mostly due to their destructive activity toward chlorophylls and other photosynthetic pigments, or the disruption of electron transport chains (e.g., juglone, sorgoleone, thymol, and chalcone).

Even if an increased level of ROS seems to be a common reaction of target plants to phytotoxins of plant origin, it is hard to find a uniform pattern of modification of the activity of cellular antioxidants. In many cases, the increased activity of CAT, SOD, and POX is detected, although this is not the rule even for the same allelochemical. Differences may be related to the target organism, the concentration of the allelochemical or duration of the treatment, as well as other conditions not mentioned by the authors in the description of the experiments.

An example of a modern view to study the MOA of allelochemicals is the transcriptome analysis (RNA-Seq) of Arabidopsis plants treated with chalcone [78]. Chalcone is a precursor of flavonoids, present in many plants in which it is responsible for their yellow coloration. The application of trans-chalcone to Arabidopsis plants led to an induction of oxidative stress and resulted in the stimulation of cell death, particularly in roots [77]. The numerous transcripts of genes involved in the response to oxidative stress and programmed cell death were up-regulated in roots of Arabidopsis [78]. Genes related to apoptosis, oxidative stress, and cell death were up-regulated in roots after a short-term treatment (1–6 h). Moreover, as suggested by the authors, the simultaneous increased expression of redox signaling and auxin-related genes in chalcone-stressed plants may indicate the occurrence of interactions between the two processes. A similar coincidence between the enhanced ROS production and auxin levels was detected in Arabidopsis roots, whose growth was inhibited by citral [52]. This seems to be a common observation, confirmed by other authors, e.g., after plant treatment with *meta*-tyrosine [73], canavanine [104], or cyanamide [71], which reveals the severe impact of redox control on the phytohormonal regulation.

The development of -omic studies results in some, but still rare, attempts to correlate the morpho-physiological effects of allelochemical action with changes in the metabolom or proteom of the acceptor plant. Such an experiment was performed by Araniti et al. [45] with Arabidopsis plants exposed to coumarin. The metabolomic analysis indicated an increase in sugars, aromatic metabolites, and amino acids in treated plants, whereas the proteomic studies revealed a down-regulation of the ROS detoxifying proteins, confirming an imbalance of ROS metabolism.

The activity of many proteins, also including enzymatic antioxidants, depends on their post-translational modifications (PTMs) [105,106]. PTMs are the fastest and earliest of plant responses to changes in the environment, thus they are of great importance in plant science, but are almost unexplored in the context of allelopathic stress. Carbonylation and nitration belong to ROS and reactive nitrogen species (RNS)-dependent PTMs, which are considered as markers of nitro-oxidative or oxidative stress [107,108,109]. Protein carbonylation is the direct oxidation of proline, lysine, arginine, and threonine. This irreversible PTM usually leads to the loss of protein function and to the degradation of oxidized proteins [109]. Increased levels of protein carbonyl groups were detected in tomato roots, whose growth was restricted by canavanine (10–50 µM), a non-proteinogenic amino acid present in the seeds of some Fabaceous plants [110]. A similar observation was conducted in a short-term response of tomato roots to *meta*-tyrosine (Table S1), a non-proteinogenic amino acid that is present in root exudates of fescue (*Festuca rubra* spp. *commutata* and *F. rubra* spp. *rubra*) plants [74]. The protein nitration level measured as a content of 3-nitro-tyrosine increased in roots of tomato plants after their treatment with *meta*-tyrosine (Table S1) [73] and fluctuated depending on the duration of the treatment, modifying the intracellular formation of RNS (mainly ONOO^−^) after canavanine application [75,110]. Detailed proteomic studies led to the identification of differentially nitrated proteins in the roots of tomatoes exposed to canavanine, among which the most interesting was monodehydroascorbate reductase (MDAR, one of the enzyme of the Foyer–Halliwel–Asada cycle). The activity of MDAR was inhibited by the phytotoxin [111]. As the negative action of *meta*-tyrosine on the growth of roots is linked to the overproduction of ROS and RNS and the stimulation of protein nitration (Table S1) [73], we identified nitrated proteins in roots of plants growing in this non-proteinogenic amino acid (Table 2). Among them, similarly after a canavanine treatment, MDAR was found. Additional proteins of interesting functions in the context of ROS, linoleate 9S lipoxygenase A and low-temperature-induced cysteine proteinase-like protein, were also recognized (Table 2). Nitration of low-temperature-induced cysteine proteinase-like protein could result in an inhibition of uncontrolled proteolysis, which could be enhanced by the elevated content of protein carbonyl groups, thus it may act as cysteine proteinase inhibitors, which are known to improve plant tolerance to stresses [112]. Lipoxygenases play a key role in plant defenses against biotic and abiotic environmental stresses [113]. Oxylipins-signaling molecules (such as jasmonic acid) are products of the action of lipoxygenases [114,115]. The putative inhibition of lipoxygenase by nitration in *meta*-tyrosine-stressed roots may result in the lowering the biosynthesis of oxylipins. Furthermore, we cannot discard that lipoxygenase inhibition could also prevent the over-production of oxylipins, because a number of biologically active oxylipins are formed nonenzymatically via the action of ROS (Eckardt 2008, and Wasternack and Feussner 2018), whose increased level is observed after the application of *meta*-tyrosine.

## 5. Conclusions

Disturbances in redox homeostasis seem to be the cause of the induction of secondary oxidative stress in the allelochemical-dependent interaction between plants. Many phytotoxins stimulate ROS production and modify cellular ROS scavenging activity in target organisms. There is no doubt that the application of most allelochemicals to acceptor plants results in oxidative damages of membranes due to enhanced lipid peroxidation. A direct impact on redox homeostasis is the MOA of only a few allelochemicals (e.g., juglone) in a special cellular environment. Generally, plant response to allelochemicals, similarly to other biotic stressors, requires a commitment of ROS as signaling molecules. The additional proofs for activation of ROS-induced transduction pathways are necessary to complete our knowledge regarding the action of ROS in this allelopathic interaction.

In addition, studies carried out on plant tissues, with the use of other plant extracts or compounds isolated from plants, may provide an important encouragement to search for the biochemical tools used for lethal modification of ROS metabolism in the cells of the acceptor plants. This could lead to the discovery of environmentally friendly herbicides, whose MOA will involve the induction of toxic nitro-oxidative stress due to the disturbances in ROS/RNS metabolism.

Authors dedicate this manuscript to the memory of Prof. Renata Bogatek (1949–2020), the founder and the leader of our group, and the pioneer of the study of the induction of oxidative stress in the allelopathic interaction between plants. 

## Figures and Tables

**Table 1:** The modification of ROS metabolism as an element of allelopathic activity of individual chemicals. The physiological effects of the phytotoxin in the acceptor plant were correlated to oxidative damages or ROS mediated/dependent processes. Structures of allelochemicals were downloaded from PubChem database (CID no), references for PubChem data are included in Supplementary Materials.

Allelochemical and Its Dose Used in the Experiment/CID	Donor Plant	Acceptor Plant	Physiological Effects in the Acceptor Plant	Modification of ROS Metabolism in the Acceptor Plant	Literature
ß-Cembrenediol (50–800 µM) 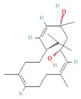 CID 6440192	Tobacco (*Nicotiana tabacum* L.)	Lettuce (*Lactuca sativa* L.)	Reduction of root and shoot growth, induction of cell death, restriction of cell division (mitotic index), increased MDA level, and increased proline content	Increased ROS level	[43]
Isoliquiritigenin—halcone type flavonoid (600–1000 µM) 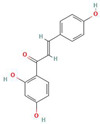 CID 638278	Chinese liquorice (*Glycyrrhiza uralensis* Fish.)	Lettuce (*Lactuca sativa* L.)	Inhibition of root growth, decreased cell viability, induction of cell death, increased MDA level, and increased proline content	Increased ROS level	[44]
Coumarin (100 µM) 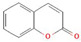 CID 323	*Araliales*, *Rutales*, *Asterales*, *Fabales*, *Oleales*, *Urticales*, *Thymelaeales*, *Apiaceae* (*Umbelliferae*), *Rutaceae*, *Asteraceae* (*Compositae*), *Fabaceae* (*Leguminosae*), *Oleaceae*, *Moraceae*, *Thymelaeaceae*	Arabidopsis (*Arabidopsis thaliana* (L.) Heynh.)	Inhibition of growth and photosynthesis rate, increased MDA content, increased electrolyte leakage, and decreased membrane stability	Increased H_2_O_2_ level, decreased content of proteins linked to ROS metabolism: 2-Cys peroxiredoxins and peroxidases	[45]
Coumarin (1000 μM) 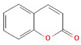 CID 323		Wheat (*Triticum aestivum* L.) aleurone layer	Increased cell death and induction of α-amylase activity	Increased ROS level and decreased SOD, CAT, and APX activity	[46]
Coumarin (Umbelliferone) (1–400 µM) 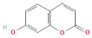 CID 5281426	Stellera (*Stellera chamaejasme L*.)	Lettuce (*Lactuca sativa* L.)	Inhibition of root and shoot growth, loss of cell viability, arrested cell division, increased proline content, and increased MDA level	Increased ROS production and increased H_2_O_2_ level	[47]
ß-pinene (10–100 µM) 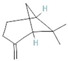 CID14896	Many aromatic plant species—common producers of essential oils	Wheat (*Triticum aestivum* L.)	Inhibition of root and shoot growth, increased MDA level, increased lipid peroxidation, increased electrolyte leakage, and increased lipooxygenases activity	Increased O_2_^•−^ and H_2_O_2_ level, and increased activity of SOD, CAT, APX, and GPX	[48]
ß-pinene (20–800 µg mL^−1^) 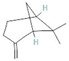 CID 14896		Rice (*Oryza sativa* L.)	Reduced growth of roots and coleoptiles.	Increased POX and polyphenol oxidase activity	[49]
ß-myrcene (70–700 µg mL^−1^) 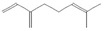 CID 31253	Many aromatic plant species—common producers of essential oils, e.g., verbena (*Verbena officinalis* L.), lemongrass (*Cymbopogon* sp.), hops (*Humulus lupulus* L.), thyme (*Thymus* sp.), and rosemary (*Rosmarinus officinalis* L.)	Weeds: wild oat (*Avena fatua* L.), purple nutsedge (*Cyperus rotundus* L.), and small canary grass (*Phalaris minor* Retz.)	Inhibition of seed germination, growth of seedlings, decreased cell viability, increased electrolyte leakage, MDA increased level, and increased membrane damage	Increased H_2_O_2_ content	[50]
ß-myrcene 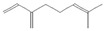 CID 31253	Many aromatic plant species—common producers of essential oils, e.g, common sage (*Salvia officinalis* L.) and mugwort (*Artemisia* sp.)	Rice (*Oryza sativa* L.)	Inhibition of root growth, increased lipooxygenases activity and gene expression, up-regulation of gene encoding transcription factor WRKY71(defense related genes). Decreased transcripts level of CycA1, CycB1 and CycD1	Increased ROS production, increased activity of SOD, and decreased POX activity	[51]
Citral (194–311 μM) 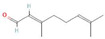 CID 638011	Many aromatic plant species—common producers of essential oils, e.g., myrtle trees, basil, lemon, lime, lemongrass, orange, and bergamot	Arabidopsis (*Arabidopsis thaliana* (L.) Heynh.)	Inhibition of root growth, microtubule disruption, alteration in cell division, and drastic fall in mitotic activity	Increased H_2_O_2_ level	[52]
Juglone 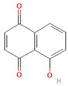 CID 3806	Black walnut (*Juglans nigra* L.)	Bur clover (*Medicago polymorpha* L.) and hop clover (*Medicago lupulina* L.)	Chlorosis, decreased levels of transcripts of WRKY53, particularly in *M. polymorpha*, which is more sensitive to juglone	Increased activity and transcript levels of CAT, increased activity of APX and POX, and increased total glutathione and GSH content	[53]
Juglone (20 µM) 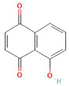 CID 3806	Black walnut (*Juglans nigra* L.)	*Chlamydomonas reinhardtii*	Decreased level of photosynthetic pigments (chlorophylls and carotenoids)	Increased O_2_^•−^ formation and decreased content of tocopherol	[54]
Juglone 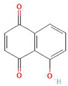 CID 3806	Black walnut (*Juglans nigra* L.)	Maize (*Zea mays* L.)		Increased H_2_O_2_ generation and increased CAT and SOD activity	[55]
Juglone 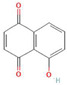 CID 3806	Black walnut (*Juglans nigra* L.)	Maize (*Zea mays* L.)		Increased activity of CAT and SOD	[56]
Juglone 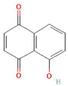 CID 3806	Black walnut (*Juglans nigra* L.)	Lettuce (*Lactuca sativa* L.)	Increased lipid peroxidation	Increased levels of H_2_O_2_, O_2_^•−^	[57]
Gallic acid (50 µM) 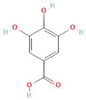 CID 37	Common reed (*Phragmites australis* (Cav.)Trin. Ex Steud) (as an invasive plant)	Arabidopsis (*Arabidopsis thaliana* (L.) Heynh.)	Inhibition of root growth and microtubule disruption	Increased ROS production (staining with H_2_DCF-DA)	[58]
Thymol (300 µM) 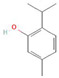 CID 6989	Species belonging to *Labiateae* family— common producers of essential oils.	Arabidopsis (*Arabidopsis thaliana* (L.) Heynh.)	Inhibition of plant growth, decreased content of chlorophyll A and carotenoids, inhibition of photosynthesis, and increased MDA level	Increased H_2_O_2_ level, increased content of proteins linked to ROS detoxification, probable peroxidase 26, superoxidase dismutase [Fe] 1 chloroplastic, and thioredoxins	[59]
Pyrogallic acid (50 mg/L) 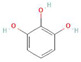 CID 1057	Submerge macrophytes.	Cyanobacteria (*Microcystis aeruginosa* Kützing)	Reduction of phytoplancton density	Increased O_2_^•−^ generation and H_2_O_2_ levels, decreased activity of SOD under high concentrations, and increased activity of SOD under low concentrations.	[60]
Benzoic acid (2.5–5.0 mM) 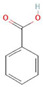 CID 243	Peach (*Prunus persica* (L.) Batsch) plants exhibiting autotoxic activity	Peach (*Prunus persica* (L.) Batsch)	Inhibition of growth, inhibition of photosynthesis, decreased content of chlorophyll, and increased MDA level	Increased activity of SOD, CAT, and POX to 2 weeks of treatment, declined after 30 days.	[61]
Ferulic acid (8–20 mM) 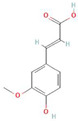 CID 445858	Many plants, e.g., cucurbitaceae, also of strong autotoxic activity	Cotton (*Gossypium* sp.)	Inhibition of growth, decreased membrane fluidity, and destruction of mitochondrial function	Increased H_2_O_2_ content, increased O_2_^•−^ generation, and decreased activity of CAT, SOD, and POX	[62,63]
Ferulic acid (1 mM) 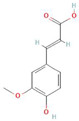 CID 445858		Soybean (*Glycine max* (L.) Merr.)	Inhibition of root growth, disintegration of root cup, and increased lignin content	Decreased H_2_O_2_ content and increased POX activity	[64]
Nerolidol (50–800 µM) 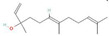 CID 5284507	Producer of essential oils	Arabidopsis (*Arabidopsis thaliana* (L.) Heynh.)	Root growth inhibition and malformation, increased content of IAA, and increased MDA level	Increased level of H_2_O_2_ and increased activity of CAT and SOD	[65]
Rosmarinic acid (175 µM) 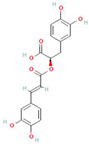 CID 5281792	Species of Boragianaceae and Lamianaceae families	Arabidopsis (*Arabidopsis thaliana* (L.) Heynh.)	Root growth inhibition and malformation, induction of cell death, mitochondrial disorganization, and increased number of divided mitochondria	Increased H_2_O_2_ and O_2_^•−^ levels, and decreased activity of SOD and CAT	[66]
Farnesene (.....) 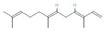 CID 5281516	Producer of essential oils	Arabidopsis (*Arabidopsis thaliana* (L.) Heynh.)	Root growth inhibition and malformation, disruption of root gravitropism, and alterations of the microtubule organization	Increased H_2_O_2_ and O_2_^•−^ levels	[67]
2–3H-benzoxazolinone (BOA) (0.1–3 mM) 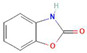 CID6043	Crop plants, e.g., wheat, maize, and rye (*Secale cereale* L.)	Arabidopsis (*Arabidopsis thaliana* (L.) Heynh.)	Inhibition of root growth, delay in cell division, chlorosis, necrosis, and increased MDA level	Increased level of H_2_O_2_ after long-term treatment	[68]
2–3H-benzoxazolinone (BOA) (5 mM) 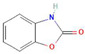 CID6043		Mung bean (*Phaseolus aureus* L.)	Growth inhibition, increased lipid peroxidation, and increased MDA level	Increased level of H_2_O_2_ and increased activity of SOD, APX, GPX, CAT, and GR	[69]
Cyanamide (1.2 mM–10 mM) 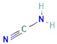 CID 9864	Hairy vetch *Vicia villosa* Roth	Maize (*Zea mays* L.), onion (*Allium cepa* L.), and tomato (*Solanum lycopersicum* L.)	Inhibition of root growth, restriction in cell division, increased MDA content, and increased content of IAA and ethylene emission	Increased levels of H_2_O_2_ and O_2_^•−^	[70,71,72]
*meta*-tyrosine (50–250 µM) 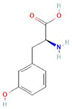 CID 6950578	Fescue (*Festuca rubra L.*)	Tomato (*Solanum lycopersicum* L.).	Root growth inhibition and malformation, and increased phenolics content	Increased levels of H_2_O_2_ and O_2_^•−^; down-regulated expression of *CAT1*, *CAT2*, and CAT3; and increased protein oxidation and nitration level	[73,74,75]
Chalcone (20–73 µM) 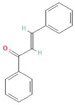 CID 637760	Widely distributed in various plant species	Arabidopsis (*Arabidopsis thaliana* (L.) Heynh.)	Root and shoot growth inhibition, de-greening of the shoots (decreased content of chlorophylls), disorders in chloroplast structure with deformed thylakoids, and abnormalities in mitochondria structure in roots	Increased levels of H_2_O_2_, decreased activity of POX, genes encoding POX down regulated in roots genes	[76,77,78]

**Table 2:** MALDI MS/MS identification after trypsin in-gel digestion of the nitrated proteins of tomato roots treated with *meta*-tyrosine (50 and 250 µM) for 24 or 72 h.

	Culture Period (h)	Description
*meta*-Tyrosine 50 µM	24	Luminal-binding protein 5
		Phosphoglycerate kinase,
		Prohibitin-1, mitochondrial-like
		Vivilin precursor (fragment)
	72	Polyphenol oxidase D, chloroplastic
		Prohibitin-3 mitochondrial
		Monodehydroascorbate reductase
		Glucan endo-1,3-beta-glucosidase B precursor
*meta*-Tyrosine 250 µM	24	Luminal-binding protein 5
		Linoleate 9S-lipoxygenase A
		Vivilin precursor (fragment)
	72	Glucan endo-1,3-beta-glucosidase B precursor
		Prohibitin-3 mitochondrial
		Monodehydroascorbate reductase
		Phospoglycerate kinase
		Low-temperature-induced cysteine proteinase-like protein

## Supplementary Materials

The following are available online at https://www.mdpi.com/article/10.3390/antiox10111648/s1. Details of the database search and the reference list of the structures of allelochemicals. Table S1. Length of the roots of tomato seedlings supplemented with *m*-Tyr (50, 250 μM) for 24 or 72 h and the level of ROS (H_2_O_2_, O_2_^•−^), RNS (NO, ONOO^−^), 3-nitrotyrosine in roots of plants treated with *m*-Tyr.
