# Supplementary material for: ROS Metabolism Perturbation as an Element of Mode of Action of Allelochemicals
Source: Antioxidants (Basel). 2021 Oct 20;10(11):1648. doi: 10.3390/antiox10111648 (PMC8614981; doi:10.3390/antiox10111648)
Supplement: Supplementary file 1 [file antioxidants-10-01648-s001.zip › antioxidants-1413919-supplementary.pdf]

Supplementary Materials

ROS Metabolism Perturbation as an Element of Mode of Action of Allelochemicals

Pawel Staszek, Urszula Krasuska, Katarzyna Ciacka and Agnieszka Gniazdowska

Database Search

In July 2021, a literature search was performed with the Web of Science (WoS) online database (https://www.webofscience.com/) to identify papers with the following search strategy:

1. Search for papers on allelopathy: ("allelopathy" OR "allelochemical*"). This query searched for papers that contain the word allelopathy/ allelochemical and its derivatives in their title, abstract, or keywords.
2. Search for papers on oxidative stress in plants: ("oxidative+stress" AND "plant*"). This query searched for papers that contain the word oxidative stress and plant with its derivatives in their title, abstract, or keywords.
3. Search for papers on ROS in allelopathy: (("allelopathy" OR "allelochemical*") AND ("reactive+oxygen+species" OR "ROS" OR "oxidative+stress" OR "oxidative+damage")). This query searched for papers that contain the word allelopathy or allelochemical with its derivatives and reactive oxygen species or ROS or oxidative stress or oxidative damage in their title, abstract, or keywords.
4. Search for papers mode of action of allelchemicals: (("allelopathy" OR "allelochemical*") AND ("mode+of+action" OR "allelochemical+target" OR "phytotoxicity+mechanism* " OR "signal+transduction")). This query searched for papers that contain the word allelopathy or allelochemical with its derivatives and mode of action or allelochemical target or phytotoxicity mechanism with its derivatives or signal transduction in their title, abstract, or keywords.

Articles published until December 31, 2020 were analysed. No restrictions were imposed on publication type (e.g., original article, review, and editorial), or publication language.

Destabilization of Reactive Oxygen (ROS) and Reactive Nitrogen Species (RNS) Metabolism by *meta*-Tyrosine

meta-Tyrosine (*m*-Tyr) is a structural analogue of a proteinogenic amino acid - phenylalanine. *m*-Tyr as a strong allelochemical suppressed growth of plants of over 50 species, both weeds and crops [1]. In the most recent studies conducted on tomato (*Solanum lycopersicum* L.) seedlings we have demonstrated that *m*-Tyr (50, 250 μM) modified metabolisms of ROS and RNS [2–4]. The first observed morphological effect of *m*-Tyr supplementation was inhibition of elongation growth of roots of young seedlings (Tab. A1). It was accompanied by increased content of H_2O_2 and stimulated $O_2^{\bullet-}$generation (Tab. A1) [2,3]. Nitric oxide (NO) level was enhanced by 50 μM *m*-Tyr, while 250 μM *m*-Tyr supplementation decreased NO emission from roots (Tab. A1)[2]. Pattern of peroxynitrite ($ONOO^-$) generation in roots of seedlings treated with *m*-Tyr was similar to $O_2^{\bullet-}$ production pattern (Tab. A1)[4]. Alterations in 3-nitrotyrosine content corresponded to changes in $ONOO^-$ level (Tab. A1)[5].

Formation of 3-nitrotyrosine in protein structure could alter their activity. Thus, the aim of the experiment conducted on roots of tomato seedlings exposed to *m*-Tyr (50, 250 μM) for 24 or 72 h was to identify nitrated proteins that differ than these found in roots of control seedlings, growing in water.

Due to the induction of nitro-oxidative stress in the roots of tomato seedlings supplemented with *m*-Tyr, we hypothesize that some of nitrated proteins are elements of cellular antioxidant system and/or components of ROS-dependent signal transduction pathways.

Table 1. Length of the roots of tomato seedlings supplemented with *m*-Tyr (50, 250 μM) for 24 or 72 h and the level of ROS (H_2O_2, $O_2^{\bullet-}$), RNS (NO, $ONOO^-$), 3-nitrotyrosine in roots of plants treated with *m*-Tyr. Data in the table are based on data presented by Krasuska et al. [2], Andrzejczak et al. [3] converted and expressed as **percent of the control** (seedlings grown in water).

Plant Treatment	Roots Length		H_2O_2,		$O_2^{\bullet-}$		NO		$ONOO^-$		3-nitrotyrosine	
	24 h	72 h	24 h	72 h	24 h	72 h	24 h	72 h	24 h	72 h	24 h	72 h
m-Tyr 50 μM	56	35	131	136	121	139	149	109	121	112	135	109
m-Tyr 250 μM	45	13	136	162	225	141	46	60	149	132	160	163

Mass spectrometry analyses of proteins

Analyses of proteins was performed exactly as is described in details by Staszek et al [6]. Briefly, proteins from ~~form~~ roots (2 g) were extracted with 5 ml 0.1 M Tris-HCl buffer (pH 7.0) with 1% (w/v) Triton X-100, 2% (w/v) glycerol, 2 mM DTT, 0.15 M NaCl, 1% (v/v) protease inhibitor cocktail (Sigma-Aldrich) and 5% (w/v) PVPP. Protein extracts were concentrated and desalted with Pierce™ Protein Concentrator PES, 3K MWCO (Thermo Scientific™). Immunoprecipitation was done with Monoclonal anti-nitrotyrosine (Agrisera, AS10 706-100) antibodies and protein G agarose (Thermo Scientific™). After purification nitrated proteins were separated on 10% polyacrylamide gels with SDS. Protein bands of nitrated proteins which differentiate between control and *m*-Tyrosine treated were cut out, destained, digested with trypsin and peptides were extracted and lyophilized. MS/MS analysis of extracted proteins was performed with a Thermo LTQ XL. As a matrix served α-cyano-4-hydroxy cinnamic acid, peptides was dissolved in a mixture of 10% (v/v) acetonitrile and 90% of 0.1 % trifluoroacetic acid. For an identified protein at least 2 peptides were confirmed by MS/MS analysis. A database search was performed using the MASCOT search engine (Matrix-Science). Peptide tolerance was ±1.2 Da and MS/MS tolerance 0.5 Da.

References

1. Bertin, C.; Weston, L.A.; Huang, T.; Jander, G.; Owens, T.; Meinwald, J.; Schroeder, F.C. Grass roots chemistry: *meta*-Tyrosine, an herbicidal nonprotein amino acid. *Proc. Natl. Acad. Sci.* **2007**, *104*, 16964–16969, doi:10.1073/pnas.0707198104.

2. Krasuska, U.; Andrzejczak, O.; Staszek, P.; Borucki, W.; Gniazdowska, A. *meta* -Tyrosine induces modification of reactive nitrogen species level, protein nitration and nitrosoglutathione reductase in tomato roots. *Nitric Oxide* **2017**, *68*, 56–67, doi:10.1016/j.niox.2016.10.008.

3. Andrzejczak, O.; Krasuska, U.; Olechowicz, J.; Staszek, P.; Ciacka, K.; Bogatek, R.; Hebelstrup, K.; Gniazdowska, A. Destabilization of ROS metabolism in tomato roots as a phytotoxic effect of *meta*-tyrosine. *Plant Physiol. Biochem.* **2018**, *123*, 369–377, doi:10.1016/j.plaphy.2017.12.024.

4. Staszek, P.; Gniazdowska, A. Peroxynitrite induced signaling pathways in plant response to non-proteinogenic amino acids. *Planta* **2020**, *252*, 5, doi:10.1007/s00425-020-03411-4.

5. Staszek, P.; Weston, L.A.; Ciacka, K.; Krasuska, U.; Gniazdowska, A. L-Canavanine: How does a simple non-protein amino acid inhibit cellular function in a diverse living system? *Phytochem. Rev.* **2017**, *16*, 1269–1282, doi:10.1007/s11101-017-9536-y.

6. Staszek, P.; Krasuska, U.; Otulak-Kozieł, K.; Fettke, J.; Gniazdowska, A. Canavanine induced decrease in NO synthesis alters activity of antioxidant system but does not impact GSNO catabolism in tomato roots. *Front. Plant Sci.* **2019**, *10*, 1077, doi:10.3389/FPLS.2019.01077.

Reference list of structures of allelochemicals

National Center for Biotechnology Information (2021). PubChem Compound Summary for CID 6440192, beta-Cembrenediol. Retrieved September 19, 2021 from https://pubchem.ncbi.nlm.nih.gov/compound/beta-Cembrenediol.

National Center for Biotechnology Information (2021). PubChem Compound Summary for CID 638278, Isoliquiritigenin. Retrieved September 19, 2021 from https://pubchem.ncbi.nlm.nih.gov/compound/Isoliquiritigenin

National Center for Biotechnology Information (2021). PubChem Compound Summary for CID 323, Coumarin. Retrieved September 19, 2021 from https://pubchem.ncbi.nlm.nih.gov/compound/Coumarin

National Center for Biotechnology Information (2021). PubChem Compound Summary for CID 5281426, Umbelliferone. Retrieved September 19, 2021 from https://pubchem.ncbi.nlm.nih.gov/compound/Umbelliferone

National Center for Biotechnology Information (2021). PubChem Compound Summary for CID 14896, beta-Pinene. Retrieved September 19, 2021 from https://pubchem.ncbi.nlm.nih.gov/compound/beta-Pinene

National Center for Biotechnology Information (2021). PubChem Compound Summary for CID 31253, Myrcene. Retrieved September 19, 2021 from https://pubchem.ncbi.nlm.nih.gov/compound/Myrcene

National Center for Biotechnology Information (2021). PubChem Compound Summary for CID 638011, Citral. Retrieved September 19, 2021 from https://pubchem.ncbi.nlm.nih.gov/compound/Citral.

National Center for Biotechnology Information (2021). PubChem Compound Summary for CID 3806, Juglone. Retrieved September 19, 2021 from https://pubchem.ncbi.nlm.nih.gov/compound/Juglone

National Center for Biotechnology Information (2021). PubChem Compound Summary for CID 370, Gallic acid. Retrieved September 19, 2021 from https://pubchem.ncbi.nlm.nih.gov/compound/Gallic-acid

National Center for Biotechnology Information (2021). PubChem Compound Summary for CID 6989, Thymol. Retrieved September 19, 2021 from https://pubchem.ncbi.nlm.nih.gov/compound/Thymol

National Center for Biotechnology Information (2021). PubChem Compound Summary for CID 1057, Pyrogallol. Retrieved September 19, 2021 from https://pubchem.ncbi.nlm.nih.gov/compound/Pyrogallol

National Center for Biotechnology Information (2021). PubChem Compound Summary for CID 243, Benzoic acid. Retrieved September 19, 2021 from https://pubchem.ncbi.nlm.nih.gov/compound/Benzoic-acid

National Center for Biotechnology Information (2021). PubChem Compound Summary for CID 445858, Ferulic acid. Retrieved September 19, 2021 from https://pubchem.ncbi.nlm.nih.gov/compound/Ferulic-acid

National Center for Biotechnology Information (2021). PubChem Compound Summary for CID 5284507, Nerolidol. Retrieved September 19, 2021 from https://pubchem.ncbi.nlm.nih.gov/compound/Nerolidol

National Center for Biotechnology Information (2021). PubChem Compound Summary for CID 5281792, Rosmarinic acid. Retrieved September 19, 2021 from https://pubchem.ncbi.nlm.nih.gov/compound/Rosmarinic-acid

National Center for Biotechnology Information (2021). PubChem Compound Summary for CID 5281516, alpha-Farnesene. Retrieved September 19, 2021 from https://pubchem.ncbi.nlm.nih.gov/compound/alpha-Farnesene

National Center for Biotechnology Information (2021). PubChem Compound Summary for CID 6043, 2-Benzoxazolinone. Retrieved September 19, 2021 from https://pubchem.ncbi.nlm.nih.gov/compound/2-Benzoxazolinone

National Center for Biotechnology Information (2021). PubChem Compound Summary for CID 9864, Cyanamide. Retrieved September 19, 2021 from https://pubchem.ncbi.nlm.nih.gov/compound/Cyanamide

National Center for Biotechnology Information (2021). PubChem Compound Summary for CID 6950578, 3-Hydroxy-L-phenylalanine. Retrieved September 19, 2021 from https://pubchem.ncbi.nlm.nih.gov/compound/3-Hydroxy-L-phenylalanine

National Center for Biotechnology Information (2021). PubChem Compound Summary for CID 637760, Chalcone. Retrieved September 19, 2021 from https://pubchem.ncbi.nlm.nih.gov/compound/Chalcone
